# Contentment is not consent: capacity, the subjective element of Article 5, and the unequal liberty of disabled people

**DOI:** 10.1093/medlaw/fwag042

**Published:** 2026-11-05

**Authors:** Craig Wells

**Affiliations:** Kent Law School, University of Kent, Eliot College Extension, Canterbury, CT2 7NS, United Kingdom

## Abstract

In *A Reference by the Attorney General for Northern Ireland* [2026] UKSC 16, the Supreme Court invoked the 1966 Practice Statement to overrule *Cheshire West*, holding that an adult who lacks the mental capacity to decide upon their care arrangements may nonetheless give valid consent to confinement, for the purposes of the subjective element of *Storck v Germany*, by manifesting contentment with those arrangements. This article argues that the decision is wrong twice over. Conceptually, it commits a category error: across the law, consent is a communicated legal power conditioned upon capacity, and contentment is a mental state which exercises nothing. Normatively, it produces unequal protection: the conditions upon which a capacitous person may be taken outside Article 5 are policed with rigour, while a disabled person may now be removed from its protection upon an inference about their mood, drawn by those who confine them. The article reads the Strasbourg tacit-acceptance cases as capacity-sensitive, examines the court’s selective re-evaluation of MIG, MEG, and P, and answers the defences of workability and residual protection. The cure for *Cheshire West’*s burdens was a better scheme of safeguards, not the evisceration of consent.

## I. Introduction

In *Cheshire West*, Lady Hale gave the law of liberty one of its few unforgettable sentences: ‘a gilded cage is still a cage’.^1^ In *A Reference by the Attorney General for Northern Ireland*, the Supreme Court has not so much disputed the aphorism as dissolved its subject.^2^ After the *Reference*, whether the gilded enclosure is a cage at all turns upon the mood of its occupant. An adult who lacks the mental capacity to decide upon their care arrangements, but who seems settled, who says that they like where they live, who appears keen to return home after a trip away, is now taken to have given valid consent to their confinement; and a confinement validly consented to is no deprivation of liberty at all.^3^ As a matter of law, the cage is now a cage only if the prisoner is not smiling.

The decision is of the first importance, and its mechanics can be shortly stated. Invoking the Practice Statement of 1966, a unanimous court overruled *Cheshire West* and declared that the equation of mental incapacity with the absence of valid consent ‘is wrong’.^4^ Valid consent, for the purposes of the subjective element of *Storck v Germany*, is an autonomous Convention concept, and it asks not whether the person could decide upon their arrangements but whether they have a ‘de facto understanding of their situation and how they experience it’.^5^ A person with ‘a basic level of awareness’, sufficient to know and communicate whether they are happy or unhappy, may accordingly consent by an expression of wishes and feelings; and the Minister of Health’s draft Code of Practice, which translates that holding into a test of ‘evidence of a positive attitude to the care arrangements’, is within competence.^6^

This article argues that the court was wrong to sever consent from capacity, and wrong in two distinct ways which reinforce one another. The first error is conceptual. Across the legal system, and far beyond the law of capacity and care, valid consent is the communicated exercise of a legal power: an act with an effect upon what is lawful and unlawful between persons, performed by communicating an intention, and conditioned upon the capacity to understand what is agreed to. That description is the convergent account of the philosophical literature and, as Section III shows, the account the law gives of itself.^7^ Capacity, in short, is not a condition bolted onto consent from the outside; it is constitutive of the act, and when it is subtracted, what remains is not a thinner consent but no consent. Contentment fails every element of the description. It is a state, not a deed; a fact about how a person feels, not a decision about what others may do. To treat an inferred contentment as the valid consent which satisfies the subjective element of *Storck* is therefore not a generous application of the concept but a misdescription of it: a category error, by which the registering of a mood is mistaken for the exercise of a power.

The second error is one of equality. The mechanism by which a person may pass out of the protection of Article 5, consent, is formally the same for everyone; the conditions of its exercise are not. Where the consenting person is capacitous, the law polices the exit with rigour. Agreement may not remove the protection at all; consent extracted between greater and lesser confinements may be no real consent; and what has been given may at any moment be withdrawn.^8^ Where the person is disabled, the *Reference* installs an exit of altogether lighter construction: an inference of contentment suffices, and the body charged with drawing it is the body that confines.^9^ The threshold for losing the right to liberty is thereby set lowest for those least able to exercise the legal power to defeat it. That is a differential enjoyment of a Convention right on the ground of disability, and it inverts the principle of *HL v United Kingdom* that incapacity makes the protection of Article 5 more important, not less.^10^ The claim is developed below through Article 14 read with Article 5, with the Convention on the Rights of Persons with Disabilities as an interpretive aid.^11^

The scope and ambition of the argument should be fixed at the outset. This article is not a commentary upon the *Reference* at large. Its subject is the subjective element of Article 5 alone: the concept of valid consent to confinement, what that concept requires, and who is able to satisfy it. The judgment’s reworking of the objective element, its handling of the Practice Statement as a matter of precedent doctrine, and the devolution questions which carried the reference to the court are noticed only where they bear upon that subject.^12^ The occasion is a single decision; the argument is general. The account of consent assembled in Section III belongs to no corner of the law: it is drawn from private law theory, the law of sexual offences, medical treatment, child welfare, and the protective jurisdiction over vulnerable adults, and it returns to those fields. If the account is sound, it matters wherever the law is tempted to treat the appearance of agreement by a person without capacity as the exercise of a legal power, in the treatment of the compliant patient as much as in the confinement of the contented resident.^13^ The *Reference* is examined at length because it is the most consequential recent instance of that temptation; the conceptual claim would survive the judgment’s repeal.

The two limbs are independent, and the article is structured so that each can stand if the other falls. A reader who rejects the category error, and who accepts that contentment may after all be a species of consent, must still confront the equality limb: the stringency with which that consent is tested now differs by disability. A reader unmoved by the equality argument must still answer the conceptual one: what manner of consent is it that requires neither capacity nor an act? One boundary is drawn at the outset. The court held separately that a person ‘wholly incapable of expressing consent or objection’ is deprived of liberty in neither its objective nor its subjective element; that capability route raises questions of its own, and it is bracketed here.^14^ This article contests the consent route alone.

The argument proceeds in eight movements. Section II sets out what the *Reference* decided, at its strongest; Section III assembles the concept of consent as the law holds it everywhere else; Section IV names the category error; Section V confronts the Strasbourg tacit-acceptance jurisprudence upon which the court relied; Section VI develops the equality inversion; Section VII examines the court’s re-evaluation of MIG and MEG and its reservation upon P; Section VIII answers the court’s defences; and Section IX proposes the remedy.

## II. The reference and what it decided

The reference arose not from a dispute but from a draft. The Minister of Health for Northern Ireland proposed to issue a revised Code of Practice under section 288(4) of the Mental Capacity Act (Northern Ireland) 2016, and the Attorney General for Northern Ireland referred to the Supreme Court the question whether approving it would be invalid under section 24 of the Northern Ireland Act 1998 as incompatible with Convention rights.^15^ The Code’s innovation lay in the subjective element of deprivation of liberty, and the conventional reading of *Cheshire West* stood in its way.^16^

The framework within which it did so is orthodox. A deprivation of liberty under Article 5(1) comprises three elements: the objective confinement of a person to a restricted space for a not negligible time; the absence of valid consent, which Strasbourg calls the subjective element; and attribution to the state.^17^ The overruling was aimed at the second. The proposition the court set out to dislodge was that the ‘lack of mental capacity of an individual to consent to their care arrangements equates to lack of “valid consent”’, so that ‘an individual without such mental capacity is treated as unable to give valid consent to confinement’.^18^ This equation was never the contested ratio of *Cheshire West*: in 2014 it was common ground, and the litigation concerned the objective element alone.^19^

The court’s first move was definitional. Valid consent within the subjective element is an autonomous Convention concept, to be distinguished both from incapacity in domestic law and from the consent required to waive a Convention right.^20^ Waiver must be ‘free and fully informed’, and will ordinarily presuppose capacity; the subjective element, the court held, demands less.^21^ What it demands instead is a de facto understanding of one’s situation. A person who lacks legal capacity may yet possess a ‘basic understanding of their living circumstances’, in which case their view should be respected and ‘carries significant, indeed usually decisive weight’.^22^

The positive case rests upon what the court took to be a basic truth of experience. Whether someone

experiences the situation in which they are being kept and cared for as amounting to detention contrary to their wishes or as constituting a broadly supportive home environment where they are content to live their life is a basic aspect of human experience.^23^

This, the court continued, is ‘precisely the sort of matter’ about which the individual is ‘likely to have views which are meaningful, and which should be sought and should carry weight, even if the individual is mentally incapacitated in other respects’.^24^

The holding follows at paragraph 201, and it is the hinge of everything argued below. The *Cheshire West* equation ‘is wrong’: the absence of valid consent ‘is more concerned with a person’s de facto understanding of their situation and how they experience it’, so that a person with

a basic level of awareness and consciousness of their living arrangements … sufficient to enable them to know and communicate whether they are happy or unhappy with them … may be treated as able to give or withhold valid consent to confinement by an expression of their wishes and feelings.^25^

The draft Code translates the holding into a working test. By paragraph 2.12, a person without capacity ‘can also be regarded as consenting … if they actively express positive wishes and feelings’; it ‘is not enough that P does not express objection, there must be evidence of a positive attitude’.^26^ By paragraph 2.13, the burden rests upon those confining P to ‘explain at all times how P can be regarded as consenting’.^27^ The court also built in a limiting principle, which should be stated at full strength: ‘[w]here there is serious doubt, no inference of valid consent should be drawn’.^28^ Whether that principle can carry the weight later placed upon it is the business of Section VIII.

The boundary drawn in the introduction bears restating in the judgment’s own terms: for the person who retains basic awareness, the route out of Article 5 is consent, the manifested contentment just described; for the person ‘wholly incapable of expressing consent or objection’, neither element is present at all.^29^ The category error charged below lies in the consent route alone: in the passage from paragraphs 135 and 139 to paragraph 201 and paragraph 2.12 of the Code, where a registered mood purports to be a normative act.

Three further features of the judgment will matter later. First, the court inverted Lady Hale’s analysis. Where she had reasoned that, if a person cannot express a view, ‘the subjective element of a deprivation of liberty is established’, the court held that she ‘got the analysis on this point the wrong way round’: on its reading of Strasbourg, ‘this would indicate that it is not established’.^30^ Secondly, the court disapproved Lord Neuberger’s reasoning in *Cheshire West* that conditions which would deprive a person able to object of their liberty cannot cease to do so for a person unable to object.^31^ The combined effect is a scheme in which the only path into Article 5, for an incapacitous person, is active and manifested objection. Thirdly, the court applied its test selectively, and the application can only be understood with the three individuals whose appeals made *Cheshire West* in view. MIG and MEG were sisters with learning disabilities, settled in foster and residential placements under continuous supervision; P was a man of 38 years with cerebral palsy and Down’s syndrome, completely under the control of staff. Their situations are described more fully in Section VII. On the new test, MIG and MEG were re-evaluated and found never to have been deprived of liberty at all, while the position of P, the one individual whom the *Cheshire West* court had found deprived unanimously, was expressly reserved.^32^ Together these features frame the question this article asks: what is this thing the court calls consent, which requires neither capacity nor an act, and which operates only where confinement sits lightest?

## III. Consent across the law

Before the category error can be named, the concept must be assembled. This section gathers the law’s working understanding of consent from settings in which nothing turns on Article 5. Wherever the law takes consent seriously, the same three-part structure appears: consent is a legal power; its exercise must be communicated; and the power exists only in those with the capacity to wield it. Each element repays attention, because the consent recognized in the *Reference* will be found to fail all three.

First, consent is a legal power in the strict sense, with an effect upon what is lawful and unlawful: its exercise alters what others may permissibly do. Essert defines a legal power as the ability to change the normative situation of oneself or another ‘merely by communicating an intention’ to do so, ‘without having also to change the nonnormative facts of the world’.^33^ Consent is the paradigm. Before it is given, the surgeon’s incision is a battery, the entry to land a trespass, the locked door an imprisonment; after it, each is permitted, though not a single physical fact has changed. Dougherty makes the same point in the moral register: consent stands alongside promising as one of the ways in which persons reshape their normative relations, releasing others from duties they would otherwise owe.^34^ The reason for insisting upon this is not formalism. It is that a legal power is something *exercised*. It has a holder who wields it, an object upon which it operates, and a moment at which it is brought to bear. A state of mind, however genuine and however warm, exercises nothing.^35^

Secondly, the exercise must be communicated. ‘A private intention is insufficient’: consent must be expressed, because those whose duties it releases must be able to know where they stand.^36^ Consent is given, not found. It is an act addressed by one person to another, not a condition that an observer may detect and then attribute. An expression of feeling can of course be communicated, and the *Reference* makes much of that; but what the communication element of consent requires is the communication of a decision, the exercise itself, and not the supply of evidence from which a decision might afterwards be reconstructed by the very party who benefits from finding it.

One further feature distinguishes consent to confinement from the transactions around which the philosophical literature was built, and it raises the demands of the analysis rather than lowering them. Dougherty’s cases, like the law of informed consent from which they are drawn, concern discrete interventions: a procedure, a disclosure, a research protocol, each with a moment of agreement and a bounded object. Confinement is not an event but a state. Consent to it must therefore do something consent to an operation need not: it must persist. The person is taken to agree not once but continuously, across every day the door remains locked; and the law of liberty has always treated the continuing character of the agreement as essential, for what has been given may at any moment be withdrawn, and the confinement must yield when it is.^37^ It should be conceded at once that the communicative account was not designed for standing states, and that its extension to them requires argument; Dougherty’s critics would add that his is a demanding vision of informed consent, built for the discrete worlds of treatment and research. The argument runs against the *Reference*, not for it. A continuing consent presupposes a continuing grasp of what is being agreed to and a continuing ability to revisit it; the capacities it demands are greater than those demanded by a single signature, not smaller. A person who cannot perform the discrete act of consenting to a bounded intervention is not better placed to sustain a standing agreement to an open-ended one; and an inference of contentment, drawn at intervals by the confining body, supplies not a continuing consent but a series of guesses about a consent that was never given.

Thirdly, and decisively, the legal power belongs only to those with the capacity to exercise it, and capacity enters not as an external condition of validity but as part of what consent is. Section 74 of the Sexual Offences Act 2003 defines consent as agreement made by choice by a person with ‘the freedom and capacity to make that choice’.^38^ In *A Local Authority v JB*, the Supreme Court built the same structure into the civil law of capacity: the information relevant to the decision to engage in sexual relations is internal to the act of consenting, and the ability to understand, retain and use or weigh it is what makes the act possible at all.^39^ Two consequences of that architecture were settled long before the *Reference*, and the judgment confronts neither. The first must be stated with more care than the slogan that nobody may consent on another’s behalf, for the slogan is false as a generalization and the law’s position is differentiated by field. Parents consent on their children’s behalf every day, and an attorney or a court-appointed deputy, if so empowered, may consent to care or treatment for the person they act for. In medical treatment more broadly, substituted decision-making does real work: a best interests determination under section 4 of the Mental Capacity Act 2005 renders lawful an intervention that would otherwise be a battery, and section 5 protects those who carry it out.^40^ What that machinery supplies, however, is authorization, not consent: the statutory defence operates precisely because consent is absent, and it never pretends that the act of consenting has been performed. In the field of sexual relations, *JB* confirms that the act cannot be performed by anyone else at all.^41^ In the confinement context, the position is stricter still, and it is statutory as well as jurisprudential. Section 4A of the 2005 Act provides that the Act authorizes no one to deprive another of liberty save by court order or under the statutory schemes; an attorney or deputy, however wide the instrument, cannot supply the missing consent; and *In re D* holds that not even a parent’s consent can displace the subjective element for a 16-year-old child.^42^ The distinction matters because it is the distinction the *Reference* collapses. Substituted judgment, where the law permits it, is exercised openly, in the person’s interests, under the disciplines of section 4, and it is never mistaken for the person’s own act. The Code’s inference of consent from demeanour is the person’s own act as reconstructed by another; that is neither consent nor honest substitution. The second is that cooperation is not consent. The Mental Health Act Code of Practice states it without qualification: ‘a person who lacks capacity is unable to consent or refuse treatment, even if they co-operate with the treatment or actively seek it’.^43^ Eager cooperation, treatment actively sought, manifest contentment: none of it is consent, for what is missing is not enthusiasm but the legal power.^44^

The law does not treat the appearance of consent as self-certifying either; the more vulnerable the consenting party, the more sceptically the law examines that appearance. A parent’s agreement to a child’s accommodation under section 20 of the Children Act 1989, given under the shadow of threatened proceedings, may be no real consent at all, and what has been given may be withdrawn at any moment.^45^ A child’s ‘apparently balanced and free decision’ to accept confinement ‘may be quickly revised and/or reversed’; the authorities show ‘how insecure may be the child’s apparent consent’.^46^ Where an adult retains capacity but stands within reach of constraint, coercion or undue influence, the High Court’s inherent jurisdiction intervenes to protect a person ‘disabled from making a free choice, or incapacitated or disabled from giving or expressing a real and genuine consent’.^47^ Coggon and Miola press the same distinction in medical law: a regime that accepts the outward form of agreement while ignoring its conditions delivers a respect for self-determination that is ‘ostensible rather than substantive’.^48^ The gradient across these fields is unmistakable. Scrutiny of apparent consent rises with the vulnerability of the person said to have given it, and Section VI will hold the *Reference* against that gradient. For present purposes the conceptual point suffices: in every neighbouring field the question is never merely whether something resembling agreement occurred, but whether the conditions existed under which agreement could be the person’s own act.

The philosophical account of informed consent draws these threads together. Dougherty’s Absolute Position Principle holds that a person gives valid consent only if they occupy a sufficiently good position for deciding whether to consent, which requires that they have understood the relevant information, including the risks, the benefits and the alternative options.^49^ His *No Shared Language* case might have been written for the *Reference*: where patient and medic share no common language, valid consent is, in Dougherty’s words, ‘impossible’, however much goodwill passes between them, and the intervention must instead be justified openly by the patient’s interests.^50^ One asymmetry in the account deserves emphasis, because this article relies upon it. Dissent is not the mirror of consent. Objection has moral and legal force even where the objector could not give valid consent, for refusal does not exercise a legal power so much as decline to have one exercised over you, and respecting it demands no capacity threshold at all.^51^ Nothing in this article disturbs the weight the law gives to the objections of people who lack capacity. What is contested is the opposite manoeuvre: the conversion of non-objection’s cheerful cousin into a legal power its bearer cannot hold.

What, then, would a de facto capacity to consent to confinement require? The question has a judicial answer, and it is conspicuous that the *Reference* nowhere engages with it. In *A Primary Care Trust v LDV*, Baker J identified the information relevant to consenting to arrangements amounting to a deprivation of liberty: that she was in hospital for care and treatment of a mental disorder; that the arrangements would include varying levels of supervision, physical restraint and medication; that staff could carry out searches; and that she must seek permission to leave, would leave only under supervision, and, if she left without permission, would be found and returned, if need be, by the police.^52^ Baker J’s catalogue was compiled for a hospital, and the point travels to other settings with adjustment rather than without it. In a domestic or quasi-domestic placement of the kind occupied by MIG and MEG, the relevant information changes in content: the supervision is gentler, the boundary is a foster home’s front door rather than a ward’s, the consequence of leaving is retrieval by a carer rather than restraint by staff. What does not change is the structure. Wherever arrangements amount in fact to confinement, what falls to be understood is their restrictive core; the domesticity of a setting alters what the restrictions are, not whether they must be understood before agreement to them can be anyone’s act. Synthesizing *LDV*, *JB* and the structure assembled above, the capacity to consent to confinement requires four things, broadly mirroring the test of capacity in the Mental Capacity Act 2005, sections 2–3: (i) an understanding that the arrangements include the restrictive measures themselves, the supervision, the restraint, the bar upon leaving; (ii) an understanding that alternatives exist, and of what withholding agreement would mean; (iii) the ability to retain, use and weigh that information; and (iv) the communication of a decision addressed to those restrictions. The final element carries the point about the object of consent which the interveners pressed upon the court: any consent capable of taking arrangements outside Article 5 ‘would need to be in relation to the restrictive measures which would otherwise give rise to a deprivation of liberty, rather than to non-restrictive aspects of the care arrangements’.^53^ A person may love the garden, the routine and the keyworker, and know nothing of the locked door. Their happiness is real. It is also about something else.

This section has assembled nothing exotic. Every element is the law’s own, stated in contexts where nobody was tempted to bend it. Held against that assembly, the test announced at paragraph 201, a basic awareness of one’s living arrangements joined to an expression of contentment, is not a modest version of consent. It exercises no legal power, addresses the wrong object, and dispenses with the capacity that makes the act possible. The *Reference* does not lower the threshold of consent. It changes the subject.

## IV. The category error

The error can now be stated with precision, and the first thing to say is where it does not lie. It does not lie in the court’s premises. Grant its premises in full: how a person experiences the situation in which they are kept is a basic aspect of human experience; their views are meaningful and should carry weight.^54^ Nothing in this article disputes any of it. The error lies in the passage from those premises to the holding: from the proposition that P’s feelings *matter* to the proposition that P’s feelings are the exercise of a legal power.^55^ Held against the structure assembled in Section III, the test fails at each stage: no legal power is exercised; an inferred contentment alters nobody’s duties, because it is not an act at all. Nothing relevant is communicated: what reaches the carer is information about a feeling, from which the carer constructs a decision the person never made. Capacity, finally, is dispensed with, though capacity is what makes the act possible. What the court calls valid consent is therefore not a lower grade of the thing the law elsewhere knows by that name. It is a different thing adopting consent’s name.

The judgment’s own architecture concedes as much. The court accepted that the waiver of a Convention right requires consent which is ‘free and fully informed’, and which will ordinarily presuppose capacity; the subjective element, it held, demands less.^56^ The bifurcation is never explained, however, and it cannot be. An autonomous Convention concept may carry an autonomous threshold; it cannot carry an autonomous metaphysics. Strasbourg may decide how demanding valid consent is to be; it cannot make a feeling into an act, any more than it could make a promise out of a hope. Once the court accepted that consent in its full sense requires information, freedom and capacity, it owed an account of how the same word, a few paragraphs later, could name something requiring none of them. No account is offered. The word is simply made to do two jobs, and the second job is done by something that is not consent. The equivocation bears consequences: consent is given the exit-effect of waiver, switching off Article 5(1) and Article 5(4) together, while exempted from every condition that waiver carries.

Jackson calls the construction of agreement around an incapacitated patient a ‘sleight of hand’^57^; Donnelly has shown how readily decision-making for those who cannot contradict it slides into the construction of a narrative.^58^ Writing of a different doctrine, the ‘best interpretation of will and preferences’, Donnelly produced a description which transfers to the draft Code without remainder:

The problem is that the best interpretation standard refuses to acknowledge that there are things that we do not, and cannot, know. It forgoes epistemic humility and assumes levels of knowledge (and justifications for actions) in situations where they do not exist. And, given that the people about whom these decisions are being made will not be in position to argue otherwise, this can happen without fear of contradiction.^59^

Substitute ‘evidence of a positive attitude’ for ‘the best interpretation standard’ and no other word needs changing. When law parts company with reality, the exercise of power becomes obscured.^60^ Before the *Reference*, a person who confined an incapacitated adult exercised power, and the law said so, with everything that followed for justification and review. After it, the same power is exercised, and the law calls it agreement.

Precision also requires marking the respectable middle position, because the court’s holding is not it. Contentment matters. Series is right that there is a moral difference between the person who is positively happy in their placement and the person who merely does not object.^61^ The law before the *Reference* already gave that difference a home. Where a person lacks capacity, section 4 of the 2005 Act requires the decision-maker to consider, so far as reasonably ascertainable, the person’s wishes and feelings, beliefs and values. *Aintree* supplies the test’s orientation: welfare is to be assessed in the widest sense, and considered ‘from the patient’s point of view’, so the happier a person is in a placement, the more powerfully their wishes and feelings tell in its favour; and it would be wrong in principle to apply any automatic discount to the point of view of a person with a mental disability. The orientation is not an abstraction; it decides cases. In *Wye Valley* itself, the settled wish and religious conviction of a man without capacity prevailed over the clinical case for amputation, Peter Jackson J holding that the wishes and feelings of people with a mental disability are as important to them as they are to anyone else, and that the patient’s beliefs and values were not to be dismissed as the products of his illness.^62^ A body of Court of Protection authority now works section 4(6) in that spirit, giving ascertainable wishes and feelings weight that rises with their strength, consistency and rationality.^63^ Happiness therefore already bore upon where a person should live, upon how intrusive their regime should be, and upon how searching its review needed to be.^64^ The same is true of the demand for respect for will and preferences made by Article 12 of the UN Convention on the Rights of Persons with Disabilities, upon which the strongest disability-rights defence of the *Reference* rests. General Comment No 1 asks states to replace substituted decision-making with support for the exercise of legal capacity: accessible information, time, trusted means of communication, assistance actually to decide.^65^ Section VI argues that the Code provides none of this as a condition of the consent it finds. The present point is narrower: everything humane in the court’s premises was already accommodated, in section 4(6), in *Aintree’*s orientation, and in the supported exercise of capacity the Convention demands. What the consent label adds is only this: the disappearance of the right.

The move also has a pedigree which the judgment, though it discusses *Ferreira*, nowhere mentions. In *Ferreira*, the Intensive Care Society submitted that a compliant, well-cared-for patient should be treated as consenting to her treatment; the Court of Appeal decided the case on other grounds and left the submission where it lay.^66^ Series traces the older genealogy: the category of the ‘non-volitional’, persons treated as having no will and no wishes upon which the law could bite, whose confinement therefore required no answer, and the long exploitation of that ambiguity by systems with an interest in it.^67^ The *Reference* is the non-volitional logic refitted for a gentler age. Where the old law said that these people have no will to overbear, the new law says that their will has been exercised, and in the confiner’s favour.

The court’s best defence deserves its strongest form: that the alternative treats P’s expressions as legally inert, and that taking disabled people seriously means letting their happiness count. Section II’s positive account answers it. The law of capacity insists that the decision in question be identified before anyone asks whether P can make it or has made it; that is the discipline of *JB*, and of Baker J’s catalogue in *LDV*.^68^ The decision the *Reference* attributes to P is consent to confinement: to the supervision, the restraint, the locked door, the return if they leave. The expressions upon which the Code relies may be addressed to something else: the placement, the garden, the staff, the routine. The court has substituted an easier decision and attached the legal consequences of the harder one. A smile at the thought of sleeping in your own bed is read as an agreement to be locked in your bedroom.

Nothing in this article denies that contentment is evidence. It is evidence of wellbeing, evidence relevant to best interests, evidence bearing upon how searching the review of a placement needs to be. What is denied is constitution: that the evidence is the act of consenting. Wherever the judgment or the Code says that an expression of feeling indicates, suggests or may be regarded as consent, the same questions expose the move: consent to what, exercised how, and by whom, with what capacity?

## V. Strasbourg and the capacity-sensitive subjective element

The court’s case stands or falls on Strasbourg, for the Practice Statement was invoked on the footing that *Cheshire West* had misread the Convention. Two preliminary points frame it. First, going beyond Strasbourg is not itself an error: where the European Court has left a question open, the domestic courts may afford the greater protection, as *In re G* establishes. The judgment’s own framework concedes the corollary: the Practice Statement may ‘more readily be applied’ where Strasbourg has moved since the earlier decision, yet ‘no one contends that there has been any relevant change’ since 2014.^69^ The overruling rests entirely upon the claim that the 2014 court misread the jurisprudence as it stood. Secondly, this section tests that claim. The thesis defended is deliberately confined: not that the jurisprudence is univocal, for it is not, but that at every point where the subjective element has actually been decided, capability has done the load-bearing work, and that the *Reference* builds its contrary reading upon the two decisions in the line, *HM* and the second placement in *Mihailovs*, that will bear it least. In *De Wilde, Ooms and Versyp*, where the subjective element began, men who had voluntarily surrendered themselves into detention were held entitled to the protection of Article 5 notwithstanding:

the right to liberty is too important in a ‘democratic society’ within the meaning of the Convention for a person to lose the benefit of the protection of the Convention for the single reason that he gives himself up to be taken into detention. Detention might violate Article 5 even although the person concerned might have agreed to it.^70^

Detention, the Court added, is ‘mandatory, as opposed to contractual’, in character.^71^ At the very top of the scale, then, stands actual agreement by capacitous adults who walked through the door themselves; even that may not take a confinement outside Article 5. At its foot sits inferred contentment, a mood read off demeanour by the detaining body. Above it sits non-dissent: compliance, the absence of objection. Above that, expressed willingness; above that, communicated, capacitous, informed agreement; and at the summit, the surrendered liberty of *De Wilde*, which the Court has held may still not be enough. The ladder ranks expressions of agreement by their force, not persons. The pattern is uniform: even the top rung, actual and capacitous submission, may fail to remove the protection of Article 5, and nothing on a lower rung has ever been allowed to do what the top rung cannot. The *Reference* takes the bottom rung and gives it the exemption the Court has withheld from the top.

*Storck*, the authority from which the subjective element derives, repays a fuller statement than it usually receives. The applicant was placed by her father, at the age of 18 years, in the locked ward of a private psychiatric clinic, where she remained for some 20 months; she attempted to escape, was forcefully returned to the clinic by the police, and was medicated against her will.^72^ The Court found the objective element established and turned to consent, and two features of its answer matter here. The first is a condition about which the *Reference* has little to say: ‘assuming that the applicant had no longer been capable of consenting’, she ‘could, in any event, not be considered as having validly agreed’ to her stay in the clinic.^73^ In the very passage that founded the subjective element, the condition the Court reached for was capability. The second is the ground upon which the Court distinguished *HM v Switzerland*: not that HM was content, but that she was ‘legally capable of expressing a view’, and had been undecided.^74^ The *Reference* explains *Storck* by the applicant’s attempts to flee; the Court’s own words tie valid agreement to the capacity to give it.

*HM v Switzerland*, the case *Storck* was distinguishing, deserves the same attention, because it is the nearest Strasbourg has come to the contented resident. HM was an elderly woman, no longer able to care for herself, who was placed in a nursing home by order and over her initial objection; the home was an open institution which allowed her freedom of movement; and once there she told the Appeals Commission that she had no reason to be unhappy, and remained undecided about whether she wished to stay.^75^ The Court, citing and following *Nielsen*, found no deprivation of liberty at all: the placement was, in the Court’s words, ‘a responsible measure taken by the competent authorities in the applicant’s interests’. Everything in the reasoning that has survived turns upon her retained capability. *HL* distinguished the case on the twin grounds that HM had sufficiently expressed consent and that the regime was an open one^76^; *Storck* explained it on the ground that HM, ‘who had been legally capable of expressing a view, had been undecided’.^77^ An untidy, capability-dependent decision, resting on *Nielsen*’s parental-rights foundation, is a thin base for the redefinition of a Convention concept.

*HL v United Kingdom* built upon the same foundation, and because the modern domestic law descends from it, the case must be set out in full. HL was an autistic man of 49 years, unable to speak and assessed as lacking capacity to consent to his admission, who had lived for 3 years with paid carers when, in July 1997, he became agitated at a day centre and was taken to Bournewood Hospital. Because he did not resist, he was admitted informally under the common law doctrine of necessity rather than under the Mental Health Act 1983; he was sedated, kept under the continuous control of the clinical staff, and his carers were discouraged from visiting in case he tried to leave with them. The House of Lords held that he had not been detained at all and that, if he had, necessity justified it. Strasbourg disagreed on both counts: the professionals had exercised ‘complete and effective control over his care and movements’, and the absence of any procedural rules for his admission, or of any means by which the lawfulness of his confinement could be tested, violated Article 5(1) and Article 5(4). The ‘Bournewood gap’ so exposed was closed by the Deprivation of Liberty Safeguards, inserted into the Mental Capacity Act 2005 by the Mental Health Act 2007: the very scheme whose administrative weight the *Reference* now seeks to lighten.^78^ The *De Wilde* principle applies with all the more force, the Court held, where the person is ‘legally incapable of consenting to, or disagreeing with, the proposed action’^79^; and *HM* was again distinguished because ‘it was not established that H.M. was legally incapable of expressing a view on her position’.^80^ The *Reference* glosses ‘legally incapable’ as meaning de facto capability. The gloss concedes the architecture: the exit from Article 5 turns on capability. What it cannot supply is the further step the holding requires, that a capability to register happiness is a capability to consent to confinement; Section II stands between those propositions.

One observation about HL himself must be met, because the *Reference* dwells upon it: HL’s compliance on admission was secured by sedation, and he was maintained in a tractable state in hospital by regular sedation, so it may be said that his case speaks to the wholly incapable rather than to the contented.^81^ The observation strengthens the argument it is deployed against. HL’s calm was exactly the kind of demeanour from which the Code would now permit an inference of a positive attitude: he did not object, he made no attempt to leave, and those caring for him regarded him as settled. Strasbourg’s response was not to read his acquiescence as acceptance but to hold that his incapacity made the protection of article 5 more pressing, and to demand procedure where the domestic law had offered interpretation.^82^ A sedated calm is the limiting case of the treachery of demeanour. It is the strongest reason in the whole jurisprudence for refusing to let the reading of a face stand in for the exercise of a power.

The guardianship line then confirms what *Storck* and *HL* began. In *Stanev*, a man whose legal capacity had been partially removed was placed by his guardian in a social care home, where he was under constant supervision and needed the director’s permission to leave; his stay was long enough for him to feel the full adverse effects of the restrictions, and the objective element was established.^83^ On the subjective element, the Grand Chamber acknowledged that the wishes of a person with impaired mental faculties may sometimes validly be replaced by those of another, yet held that ‘the fact that a person lacks legal capacity does not necessarily mean that he is unable to comprehend his situation’, credited Mr Stanev’s explicit objections, and found a deprivation of liberty.^84^ In *DD v Lithuania*, the applicant’s adoptive father, appointed her guardian after a court declared her legally incapacitated, had her confined at his request; she ran away twice, protested her confinement in correspondence, and challenged the guardianship; the Court found a deprivation of liberty, holding that although she had been deprived of her legal capacity ‘she was still able to express an opinion on her situation’, and had done so.^85^ The pattern of the line, completed by *Shtukaturov*, *Kędzior* and *Stankov*, is examined below; what matters first is its direction of travel. In every case the principle that incapacity in law does not silence a person in fact was used to let the confined person into Article 5, over the head of a substitute’s consent. It was never used to find that a person without capacity had themselves consented out of it.

That leaves *Mihailovs*, the court’s strongest card, and the concession should be full before the answers are given: it is the one judgment in the line in which the tacit acceptance of a man without legal capacity was held to negative the subjective element. Mr Mihailovs’s legal capacity had been removed, and his wife appointed his guardian; from January 2002 he was confined in the Īle Centre in Īle parish, which he could not leave, and where he could not receive visitors, without his wife’s consent; he objected, persistently and by every route open to him, and Strasbourg found a deprivation of liberty.^86^ At the Īle Centre in Lielbērze, from April 2010, the analysis came out the other way: he acknowledged that it was an open institution, which he had been able to leave for periods; he had refused to move from it because he said he was satisfied with his stay; and he had approached no authority for release. The Court concluded that he had ‘tacitly agreed to stay’.^87^ Two answers limit the case. The first was given in *Cheshire West* itself: Mihailovs ‘had a level of de facto understanding which had enabled him to express his objections to his first placement’, whereas some people are not capable of expressing a view either way; a capability proved by articulate objection is precisely what the Revised Code does not require.^88^ The second is the interveners’ distinction: where Strasbourg has looked behind incapacity, the incapacity was a blanket, guardianship-style removal imposed years earlier, and looking behind it restored the person’s voice; where there is a specific, contemporaneous finding that this person cannot consent to these arrangements, as the Mental Capacity Act 2005 requires, there is no blanket to look behind, and nothing for the tacit-agreement reasoning to restore.^89^ A single tacit-acceptance holding, on facts of unusual capability and unusual openness, is the entire Strasbourg foundation for paragraph 201; it will not carry the weight.

Three authorities pressed by the interveners received a considered answer, which must be met on its own terms. In *M v Ukraine*, consent to admission is valid only where the capacity to consent, and to comprehend the consequences, has been established in a fair procedure and all necessary information provided^90^; *Stankov v Bulgaria* held that principle ‘also applicable when it comes to obtaining consent for the placement in a social care home of a person whose legal capacity is impaired’.^91^ The *Reference* reads both passages as belonging elsewhere: in *M v Ukraine*, to the justification of an established deprivation under Article 5(1)(e); in *Stankov*, to the distinct objections of time limit and victim status, which attract a waiver-type standard.^92^ The reading is partly right: *N v Romania* was a justification case throughout.^93^  *Stankov*, however, announced that it would consider victim status together with the deprivation issue, and its extension sentence speaks of obtaining consent for a placement, the very act the Revised Code regulates. A structural difficulty survives even if the partition is granted in full: on the *Reference*’s own account, Strasbourg demands fair, informed, capacity-established consent where consent justifies a detention still wrapped in the safeguards of Article 5, yet asks nothing of the kind where consent extinguishes those safeguards. A legal order that polices consent more strictly where protections survive it than where they vanish with it has its priorities inverted.

The survey could continue through *Shtukaturov*, *Kędzior* and *Stankov*: in each, the same principle credited the objections of a person whose guardian had consented for them.^94^ The principle entered the jurisprudence as a shield: it stopped guardianship regimes from silencing the confined. The only decisions in which acceptance negated the subjective element are *HM* and the second placement in *Mihailovs*, both blanket-guardianship cases. The *Reference* reforges the shield into a key that locks the door: a principle built to make objections audible now makes the absence of objection dispositive.

The judgment presses one inference further: where a person can express no view at all, the subjective element may be assessed by reference to the view of the person with authority to decide for them.^95^ The dicta acknowledge that protective frameworks of substitution exist; no Strasbourg holding has ever negatived the subjective element on the strength of a guardian’s consent for a confined adult. In *Shtukaturov* the hospital treated the confinement as voluntary precisely because the guardian had consented, and a deprivation of liberty was found; so too throughout the guardianship line. *Nielsen*, the lone substitution holding, concerned a mother’s parental rights over a 12-year-old, and the judgment ring-fences it accordingly.^96^  *In re D* speaks domestically to the same effect: parental consent could not displace the subjective element for a 16-year-old.^97^

What the *Reference* erects upon this material is the inversion already described in Section I: Lady Hale’s analysis reversed, Lord Neuberger’s reasoning disapproved.^98^ The result is a scheme reserving the protections of Article 5 for those who actively and manifestly object: the people least able to object are the people least protected.

Honesty requires the concessions to be gathered in one place. *HM* is an untidy judgment, and one which rests on *Nielsen’*s anomalous, parental-rights foundation; the second placement in *Mihailovs* is a genuine tacit-acceptance holding, limited as argued above; *Storck* itself concerned a woman whose objections were written on the record, so its conditional about capability was not strictly necessary to the result; and Strasbourg has never decided the precise question the *Reference* answers. That is a point against the court, not for it. The Practice Statement demands confidence that the earlier decision was wrong, and a jurisprudence which ties valid agreement to capability at every load-bearing joint cannot supply it.^99^ There is a final irony of method. The judgment invokes the discipline of *Ullah*, that the domestic courts should ‘keep pace with the Strasbourg jurisprudence as it evolves over time: no more, but certainly no less’.^100^  *Cheshire West*, at worst, did more, which *In re G* permits where Strasbourg has left the space open. If the reading defended in this section is right, the *Reference* now does less; and that is the half of Lord Bingham’s dictum which nothing *ever* permits.

## VI. The equality inversion

The second limb does not depend upon the first. Assume, against Section IV, that contentment is a species of consent. The question then becomes how stringently the law tests that consent before the right to liberty is lost, and whether the stringency is the same for everyone. Article 14 requires the enjoyment of Convention rights without discrimination; disability is a protected status; and discrimination includes failing to treat differently those whose situations are relevantly different.^101^ The facts fall within the ambit of Article 5. What must be examined is the differential.

Total the stringent side first, for it has been accumulating throughout this article. For the capacitous, agreement may not remove protection at all; consent between greater and lesser confinements is scrutinized for reality; consent under the shadow of proceedings may be no consent; an apparently free decision is treated as insecure and revisable; situational vulnerability summons a protective jurisdiction; and consent to a care placement must be established in a fair procedure, upon all necessary information.^102^ The pattern admits of a single description: scrutiny of consent rises with the vulnerability of the person giving it.

Now the other side. For the person who lacks capacity, the *Reference* and the Code install an exit whose entire procedural content is an inference of ‘positive attitude’, drawn by the body that confines, certified by the body that confines, with no independent person engaged to test whether the self-reporting is accurate.^103^ The gradient does not merely flatten at the bottom of the scale; it reverses. The most vulnerable receive the least scrutiny, and the people whose expressions are most liable to be misread become the only people whose expressions are permitted to extinguish the right unexamined.^104^

The court’s answer, foreshadowed at paragraphs 198 to 199 and given by Lieven J before it, is that a disabled person in a domestic setting is not in a situation analogous to a detainee’s.^105^ That answers a comparison this article does not make. The comparator is not between living arrangements; it is between persons whose consent operates to remove the protection of article 5. The capacitous voluntary patient and the incapacitous care home resident stand in the same legal doorway: each is confined, and each is said to have agreed. The mechanism is identical. Only the stringency differs, and it differs by disability. If the analogy is resisted, *Thlimmenos* supplies the alternative route. On either route, very weighty reasons are required.

The differential also inverts the principle upon which the modern law was built. *HL* holds that the protections of Article 5 matter more, not less, where the person is incapacitous.^106^ The CRPD points the same way: Article 12(2) guarantees legal capacity on an equal basis with others, and Article 14(1) the right to liberty on an equal basis with others, with disability justifying a deprivation of liberty ‘in no case’.^107^ The Supreme Court itself deployed the CRPD interpretively in *JB*, and the claim made of it in this article is modest: an instrument guaranteeing equal liberty cannot support an unequal exit from it.^108^  *ET v Moldova*, deployed by the Attorney General, does not assist either: the paragraph relied upon records the CRPD Committee’s view that a disabled person should be able to express will and preferences about where to live, and says nothing about whether such expression constitutes valid consent under Article 5.^109^

The deepest disability-rights objection runs the other way. On the abolitionist reading of Article 12 of the CRPD, the discriminatory move is this article’s premise: to insist that capacity is constitutive of consent is to deny disabled people legal agency, and the *Reference*, on that view, is an advance for will and preferences. The answer, carried forward from Section IV, is that the *Reference* does not do what article 12 asks: General Comment No 1 demands support for the exercise of legal capacity, not the attribution of agreement. The Revised Code makes no such support a condition of the consent it finds; it attributes a decision on the strength of demeanour, certified by the confiner, which is not supported decision-making but its counterfeit. Equal recognition is not honoured by deeming agreement; it is honoured by building the conditions in which agreement can be given, and by independent scrutiny where it cannot.^110^

Could the differential be justified? The candidate aims are respect for autonomy and the relief of an overloaded system, and Section VIII takes each at full strength. It is enough here that the means fail proportionality on any view, because a less discriminatory alternative lies open and always did: give contentment its full evidential weight, within best interests and review, while the right itself remains; periodic review of a settled and contented placement ‘need not be elaborate’.^111^ A rule which removes the right where adjusting its exercise would do is not a proportionate means. It is, in Bartlett’s structural sense, the oldest pattern in this field: the differential protection of disabled people’s rights, presented as solicitude.^112^

## VII. MIG, MEG, and P

Every test meets its progenitors eventually. The *Reference* applied its own to the three individuals whose cases made *Cheshire West*, and the application is the most revealing passage in the judgment. MIG, the elder, lived with a devoted foster mother, attended college, and never attempted to leave, though she would have been restrained had she tried; MEG lived in a small residential home, sometimes restrained and prescribed tranquillizing medication. MIG and MEG were re-evaluated and found never to have been deprived of liberty at all, on both the objective and the subjective element.^113^ P was different. His was a case upon which the court ‘would prefer to reserve our opinion’, being ‘not in a position specifically to overrule’ the decision concerning him, while recording ‘some sympathy’ with Munby LJ’s contrary view below.^114^

Recall who P is. He lived under continuous supervision and control; he received intervention of an intimate and physical kind, including fingers inserted into his mouth while he was restrained; his inability to consent was conceded below.^115^ On the court’s own test, his case is the first question an examiner would set. If basic awareness joined to manifested contentment yields valid consent, does a restrained man validly consent to his restraint because he seems settled between interventions? The court did not say. It re-evaluated the two appellants whose confinement sat lightest, and reserved the one whose confinement bit hardest. A test that functions only in the absence of visible coercion is not detecting consent. It is detecting the absence of visible coercion, and relabelling it.

The charge is not bad faith; it is instability. The court confronted the hardest case its new concept would have to govern, and left it unanswered. That instability has a price under the Practice Statement, whose exercise is disciplined by legal certainty: its use should leave the law clearer and surer than it found it.^116^ The *Reference* does the opposite twice over. It overrules an equation which, as the judgment itself records, ‘was simply not argued’ in 2014, yet which it holds had nonetheless hardened into binding ratio, precisely so that the Practice Statement could be deployed against it; and it leaves expressly undisturbed the actual disposition in P’s case, whose foundation it has just removed. Practitioners are left holding a test whose authors would not apply it to the paradigm.

## VIII. Answering the defences

The strongest practical case for the *Reference* is the system *Cheshire West* built, and the judgment sets out the scale: 332,455 referrals in 2023 to 2024 against 13,700 in the year before *Cheshire West*, a backlog of 123,790, and a Law Commission estimate of £2.2 billion a year for full compliance.^117^ Mostyn J’s protest in *Rochdale* spoke for many judges asked to authorize, with full solemnity, the care of people living peacefully at home.^118^ The burden was real, and freely conceded; it changes nothing, for two reasons. First, the courts confronted with the cost of the safeguards declined to price them: the protection of the human rights of the vulnerable is ‘too important and fundamental to be sacrificed on the altar of resources’, and a failure to meet the safeguards could not ‘be successfully defended simply on grounds of lack of resources’.^119^ What the law left resource-bound was the substantive side, the options available to P; the procedural half the courts refused to surrender is precisely the half the *Reference* removes.^120^ Secondly, the redefinition buys no liberty. Defining a confined person as not deprived of their liberty ‘does not reduce any restrictions on them in reality’; it ‘only removes the safeguards’.^121^ The resources objection was pressed within weeks of *Cheshire West* and answered: a gilded cage is still a cage, however expensive the gilding.^122^

The court’s second defence is made in terms: Lady Hale’s policy reason for breadth was ‘overstated’, because an individual in care has other Convention rights, most prominently Articles 2, 3, and 8, and the assessments Article 5 requires are themselves intrusive. The rights remain, and they are the fire brigade in place of the smoke alarm. Article 5’s distinctive contribution is anticipatory: periodic, independent review going ‘to the very legality’ of the confinement while it is happening.^123^ That is what *HL* found wanting in the Bournewood gap: not that L was mistreated, but that no procedure existed by which the lawfulness of his compliant confinement could be examined.^124^ The residual rights respond to harm; Article 5 exists so that harm is not the trigger for the law’s attention.

Thirdly, the limiting principle: where there is serious doubt, no inference of valid consent should be drawn.^125^ It mitigates; it does not cure, for two structural reasons. Its object is wrong: the doubt it polices is doubt about contentment, when the question that matters is consent to the restrictions, which the contented person may never have apprehended at all. Its adjudicator, too, is interested: the burden of explaining ‘how P can be regarded as consenting’ rests upon those confining P, who record their own conclusion on a form, subject to formal review once a year, with no independent person to test the certification.^126^ Lord Neuberger doubted, in *Austin*, whether those who merely comply can be treated as having consented; a consent certified by the confiner is deemed consent with a custodian.^127^ Case’s study of ‘insight’ supplies the mirror image and the warning. For a generation, an undefined, demeanour-based clinical term did decisive legal work against patients: refusal was pathologized into incapacity.^128^ ‘Positive attitude’ is the new ‘insight’: the same kind of term, working in the opposite direction, normativizing cooperation into consent, deployed by assessors whose institutional incentives all point one way: to ease the administrative burden of control.^129^ Donnelly’s standard states what the law owes instead: overt recognition of when and how power is exercised, and rigorous oversight. The Code offers certification by the interested party.^130^

The deepest defence is epistemic: P’s expressions are the best evidence of how P experiences the placement, and a law that ignores them infantilizes. The premise is granted; the relocation is resisted. Best evidence belongs where evidence belongs, in the assessment of best interests and the calibration of review, not in the consent slot, where it ceases to be evidence and becomes a legal power P never exercised. The *Reference* does not even deliver the epistemic respect it promises. Under the Code, P’s expressions are credited when positive and met with legal compulsion when negative: objection would trigger the very detention regimes the consent finding avoids, so the consent is, in the interveners’ words, ‘illusory’.^131^ Recognition that runs one way is what Reed-Berendt and Ganguli-Mitra, after Pohlhaus, call derivatization: P is an epistemic agent when, and only when, P agrees.^132^ The evidence itself is treacherous in exactly the ways the charities documented, and Section III has already catalogued: suggestibility, misread laughter, echolalia, the fawn response of the institutionalized.^133^ The court acknowledged this evidence and deferred the difficulty to the finalisation of the Code; Example B, from Mencap’s evidence, shows what was deferred. A care provider regarded a man with a significant learning disability as content in his placement while he was ‘regularly breaking objects and hitting his head on hard surfaces’, behaviour that was one of the few means he had of communicating unhappiness; the safeguards let his family be heard, and the family was right.^134^ It was the safeguards that corrected the misreading. The *Reference* makes the misreading the event that switches the safeguards off.

Two final features complete the answer. The first is the victim gap: a person deemed to have consented is no ‘victim’ for the purposes of Article 34, so the new law insulates itself from the Strasbourg correction it would otherwise invite.^135^ The judgment invoked public authorities’ inability to test an overbroad reading in Strasbourg as a reason for intervening; its own scheme creates the mirror-image gap for the individuals affected. The second is benevolence, the judgment’s emotional undertow: these are loving placements, devoted carers, supportive homes. Almost always, yes. So was the care of HL, whose carers’ devotion nobody doubted.^136^ The right to liberty has never been a comment on anyone’s kindness. It is a protection against power, including kindly power: the kind that most blurs the edges of deprivation of liberty.

## IX. The remedy, and the return of P

Nothing above defends every consequence of *Cheshire West*, and lawful narrowing was available, as the judgment itself demonstrates by performing it on the objective side. The restored multifactorial assessment is a substantial narrowing of the acid test in its own right.^137^ The Secretary of State pressed exactly that allocation: the absence of coercion and of objection belong, if anywhere, in the objective element, where they describe what is in fact done to the person; to build upon the consent route instead would carry the law further from Strasbourg, not closer.^138^ The route the court did not need is the route this article contests: every restriction left standing, and the legal description of them abolished by an inference of mood.

There is a constitutional point too. A consent-based reconstruction of the subjective element was proposed by the Joint Committee on Human Rights in 2018; the government did not adopt it, an attempt to legislate a new statutory definition failed, and Parliament instead enacted the Liberty Protection Safeguards, reforming the procedure while leaving the definition intact.^139^ The Supreme Court has now installed it through the Practice Statement, in a reference, without a factual matrix, and without the evidence base on misreading ever being tested on real facts.

The honest scheme keeps the equation and moves the discretion to where it belongs. Capacity remains constitutive of consent. Contentment remains evidence, and is given a calibrating role: the manifest, consistent contentment of a person in a settled placement is exactly what justifies lighter, less frequent, less intrusive review, for such review ‘need not be elaborate’.^140^ The smile earns a lighter touch, not a legal disappearance. Where contentment cannot safely be read, the machinery of best interests does what Dougherty’s medic must do: justify the arrangement openly by the person’s interests, under Donnelly’s discipline of overt power and rigorous oversight.^141^ Everything the court wanted, respect for feelings, proportionate process, relief for the system, is available inside that scheme. The only thing unavailable is the fiction of consent.

Then there is P, still reserved, still the test the test cannot pass. Sooner or later a case will rise on his facts. The court that decides it will face the choice the *Reference* deferred: either say that a restrained man validly consents to his restraint by his mood, or admit that contentment was never consent.^142^ This article has argued that only one of those answers can be given by a court using words to mean what they mean. A gilded cage is still a cage, and the question the law of liberty exists to ask is not whether the occupant seems to like it, but who holds the key, and who is watching the keyholder. The door of a cage should answer to law, not to the reading of a face.

## X. Conclusion

The *Reference* begins from a humane intuition and ends in a conceptual mistake. The intuition is that the happiness of disabled people is legally nothing; and it never was. The mistake was to promote that weight into a legal power, and the mistake was made twice. Conceptually, the court took a state and called it an act: across the whole of the law, consent is a communicated, capacity-conditioned legal power, and contentment, however real and however precious, exercises nothing. Normatively, the court built an exit from Article 5 whose conditions are rigorous for the capacitous and weightless for disabled people, administered by the very bodies whose power the right exists to check, inverting the rule of *HL* that incapacity calls for more protection, not less. The two failures are separable, and either suffices. The remedy asks nothing radical: restore the equation of incapacity with the absence of valid consent; let contentment do its proper work as evidence, calibrating independent review rather than extinguishing it; and pursue the relief of the system through the lawful narrowing routes that remain open. Until that is done, the law of liberty rests upon a misdescription, and the people it misdescribes are those least able to say so. Contentment is evidence, sometimes precious evidence, of how a life is going. It is not, and never was, consent.

## Funding

The author received no funding for this work.

Conflicts of interest. None declared.

## Footnotes

1
*P v Cheshire West and Chester Council; P and Q v Surrey County Council* [2014] UKSC 19, [2014] AC 896 [46] (Lady Hale) (hereafter *Cheshire West*).

2
*A Reference by the Attorney General for Northern Ireland* [2026] UKSC 16 (hereafter the *Reference*).

3 ibid [201]; Draft Revised Code of Practice, Annex 1 to the *Reference*, paras 2.12–2.13 (hereafter ‘Draft Code’).

4
*Reference* (n 2) [201]; Practice Statement (Judicial Precedent) [1966] 1 WLR 1234.

5
*Storck v Germany* (2005) 43 EHRR 6 [74] (hereafter *Storck*); *Reference* (n 2) [84], [201].

6
*Reference* (n 2) [201]; Draft Code (n 3) para 2.12.

7 Sexual Offences Act 2003, s 74; *A Local Authority v JB* [2021] UKSC 52, [2022] AC 1322 [1], [88], [109]–[110]. For the philosophical account, see Christopher Essert, ‘Legal Powers in Private Law’ (2015) 21 Legal Theory 136; Tom Dougherty, ‘Yes Means Yes: Consent as Communication’ (2015) 43 Philosophy and Public Affairs 224.

8
*De Wilde, Ooms and Versyp v Belgium* (1979–80) 1 EHRR 373 [65]; *Secretary of State for Justice v MM* [2018] UKSC 60; *Buzadji v Moldova* App no 23755/07 (ECtHR, 5 July 2016); *Williams v London Borough of Hackney* [2018] UKSC 37, [2019] AC 421.

9 Draft Code (n 3) para 2.13.

10
*HL v United Kingdom* (2004) 40 EHRR 32 [90] (hereafter *HL*).

11 European Convention on Human Rights, art 14; Convention on the Rights of Persons with Disabilities, arts 12(2) and 14(1); Jill Stavert and Colin McKay, ‘Scotland, the Convention on the Rights of Persons with Disabilities and repurposing of mental health and capacity law’ (2025) 25 Human Rights Law Review 1, 4.

12 The constitutional dimension of the decision, and the argument that the deprivation of liberty safeguards carried into modern law the protection habeas corpus gave to those who could not petition for themselves, are developed in Craig Wells, ‘Habeas Corpus by Proxy: Liberty, the Inarticulate Subject and the Deprivation of Liberty Reference’ [2027] Public Law (forthcoming).

13 The temptation is not hypothetical in medical law: see the submission of the Intensive Care Society in *Ferreira*, discussed in Section IV below.

14
*Reference* (n 2) [142], [195]–[199].

15 Mental Capacity Act (Northern Ireland) 2016, s 288(4); Northern Ireland Act 1998, s 24 and sch 10, para 34.

16
*Reference* (n 2) [41]–[51] (Lord Sales and Lady Simler, with whom Lord Reed, Lord Hodge, Lord Lloyd-Jones, Lord Stephens and Lady Rose agreed); Practice Statement (n 4).

17
*Storck* (n 5) [74]; *Reference* (n 2) [4], [120].

18
*Reference* (n 2) [12].

19
*Cheshire West* (n 1) [37], [81]; *In re D (A Child)* [2019] UKSC 42, [2019] 1 WLR 5403 [33]; Joint Submissions on behalf of Mencap, Mind and the National Autistic Society, *A Reference by the Attorney General for Northern Ireland* UKSC/2025/0042, 30 September 2025, para 63 (hereafter ‘Joint Submissions’); *Reference* (n 2) [83]–[84].

20
*Reference* (n 2) [84], [126].

21 ibid [52], [126].

22 ibid [53(ii)], [151].

23 ibid [135].

24 ibid [139].

25 ibid [201].

26 Draft Code (n 3) para 2.12.

27 ibid para 2.13.

28
*Reference* (n 2) [53(v)], [191].

29 ibid [142], [164], [166], [176]; see also [195]–[199].

30 ibid [85].

31 ibid [93]–[94], disapproving *Cheshire West* (n 1) [67]–[68].

32
*Cheshire West* (n 1); *Reference* (n 2) [70]–[79], [203]–[205].

33 Essert (n 7) 136.

34 Dougherty, ‘Yes Means Yes’ (n 7) 226.

35 The communicative account is contested: for consent as a mental attitude, see Heidi M Hurd, ‘The Moral Magic of Consent’ (1996) 2 Legal Theory 121; Larry Alexander, ‘The Ontology of Consent’ (2014) 55 Analytic Philosophy 102. Nothing turns on the dispute: on the attitudinal view too, the attitude must be directed at the relevant conduct and formed with competence, and contentment with a placement satisfies neither condition.

36 Dougherty, ‘Yes Means Yes’ (n 7) 227.

37
*De Wilde* (n 8) [65]; *Storck* (n 5) [77]; *Williams* (n 8).

38 Sexual Offences Act 2003, s 74; *JB* (n 7) [88], [109]–[110].

39
*JB* (n 7) [1], [68]–[70], [105]–[106].

40 Mental Capacity Act 2005, ss 4-5.

41
*JB* (n 7) [93]–[94] and [105]–[106].

42 Mental Capacity Act 2005, ss 4A, 11(1)–(4) and 20(7)–(13); *In re D* (n 19) [42]–[49].

43 Mental Health Act 1983 Code of Practice (Department of Health 2015) para 24.35.

44 Mental Capacity Act 2005, ss 1(3) and 4(3); Daisy Cheung, ‘Accommodating Capacity-Restoring Interventions in the Mental Capacity Act 2005’ (2026) 34 Medical Law Review fwag013, describing capacity as ‘the gatekeeper for decision-making authority as recognized by the law’. If a contented mood could already do the work of consent, the statute’s machinery of capacity restoration would have nothing to wait for.

45
*Williams* (n 8); Joint Submissions (n 19) para 33.

46
*In re T (A Child)* [2021] UKSC 35, [2022] AC 723 [161].

47
*Re SA (Vulnerable Adult with Capacity: Marriage)* [2005] EWHC 2942 (Fam), [2006] 1 FLR 867 [77] (Munby J).

48 John Coggon and José Miola, ‘Autonomy, Liberty, and Medical Decision-Making’ (2011) 70 Cambridge Law Journal 523, 523.

49 Tom Dougherty, ‘Informed Consent, Disclosure, and Understanding’ (2020) 48 Philosophy and Public Affairs 119, 148–149.

50 ibid 139–140.

51 ibid 140.

52
*A Primary Care Trust v LDV* [2013] EWHC 272 (Fam) [39] (Baker J), quoted at Joint Submissions (n 19) fn 11.

53 Joint Submissions (n 19) para 40.

54
*Reference* (n 2) [135], [139].

55 ibid [201]; Draft Code (n 3) para 2.12.

56
*Reference* (n 2) [52], [126].

57 Emily Jackson, ‘From “Doctor Knows Best” to Dignity: Placing Adults Who Lack Capacity at the Centre of Decisions About Their Medical Treatment’ (2018) 81 Modern Law Review 247, 274.

58 Mary Donnelly, *Healthcare Decision-Making and the Law: Autonomy, Capacity and the Limits of Liberalism* (CUP 2010) 186, 209.

59 Mary Donnelly, ‘Best Interests in the Mental Capacity Act: Time to Say Goodbye?’ (2016) 24 Medical Law Review 318, 326–327.

60 ibid 327.

61 Lucy Series, *Deprivation of Liberty in the Shadows of the Institution* (Bristol University Press 2022) 232.

62
*Wye Valley NHS Trust v B* [2015] EWCOP 60 [10]-[14] and [39]-[46] (Peter Jackson J).

63
*ITW v Z* [2009] EWHC 2525 (Fam) [35]; *Re M (Best Interests: Deprivation of Liberty)* [2013] EWCOP 3456 [32] and [38].

64 Mental Capacity Act 2005, s 4(6); *Aintree University Hospitals NHS Foundation Trust v James* [2013] UKSC 67, [2014] AC 591 [39], [45] (Lady Hale); *Wye Valley* (n 62) [11].

65 Committee on the Rights of Persons with Disabilities, General Comment No 1 (2014): Article 12, Equal Recognition before the Law (CRPD/C/GC/1) paras 15–19. The abolitionist reading of art 12, and the *Reference*’s failure to meet it, are examined in Section VI.

66
*R (Ferreira) v HM Senior Coroner for Inner South London* [2017] EWCA Civ 31, [2018] QB 487, 492; the submission was not adopted: see [73].

67 Series (n 61) 171, 173.

68
*JB* (n 7) [68]–[70]; *LDV* (n 52) [39].

69
*In re G (Adoption: Unmarried Couple)* [2008] UKHL 38, [2009] AC 173; *R (Elan-Cane) v Secretary of State for the Home Department* [2021] UKSC 56, [2023] AC 559; *Reference* (n 2) [46]-[47].

70
*De Wilde* (n 8) [65].

71 ibid.

72
*Storck* (n 5) [12]–[19] and [74]–[75].

73 ibid [76]; cf *Reference* (n 2) [158].

74
*Storck* (n 5) [77].

75
*HM v Switzerland* (2004) 38 EHRR 17 [17]–[21] and [40]–[49], quotation at [48]; discussed in the *Reference* (n 2) [124] and [150].

76
*HL* (n 10) [93]; *Reference* (n 2) [156].

77
*Storck* (n 5) [77].

78
*R v Bournewood Community and Mental Health NHS Trust, ex p L* [1999] 1 AC 458 (HL); *HL* (n 10) [9]–[13], [89]–[94], [116]–[124], and [135]–[142], quotation at [91]; Mental Health Act 2007, s 50 and sch 7, inserting sch A1 into the Mental Capacity Act 2005.

79
*HL* (n 10) [90]; cf *Reference* (n 2) [155].

80
*HL* (n 10) [93]; *Reference* (n 2) [156].

81
*Reference* (n 2) [152]; *HL* (n 10) [9]–[12] and [46].

82
*HL* (n 10) [90] and [120]–[121].

83
*Stanev v Bulgaria* (2012) 55 EHRR 22 [124]–[129]; *Reference* (n 2) [162].

84
*Stanev* (n 83) [130]; *Reference* (n 2) [163].

85
*DD v Lithuania* [2012] MHLR 209 [145]–[150], quotation at [150]; *Reference* (n 2) [165]–[166].

86
*Mihailovs v Latvia* [2014] MHLR 87 [128]–[137]; discussed in the *Reference* (n 2) [170]–[172].

87
*Mihailovs* (n 86) [138]–[140].

88
*Cheshire West* (n 1) [55]; *Reference* (n 2) [85].

89 Joint Submissions (n 19) paras 17 and 24.

90
*M v Ukraine* [2013] MHLR 255 [77]; *Reference* (n 2) [168]–[169].

91
*Stankov v Bulgaria* App no 25820/07 (ECtHR, 17 March 2015) [89]–[91].

92
*Reference* (n 2) [177]–[179]; cf Joint Submissions (n 19) para 28.

93
*N v Romania* [2018] MHLR 288 [165]; *Červenka v Czech Republic* [2017] MHLR 195 [102]–[104]; *Reference* (n 2) [180]–[181].

94
*Shtukaturov v Russia* (2012) 54 EHRR 27 [107]–[109]; *DD* (n 85) [150]; *Kędzior v Poland* [2013] MHLR 115 [58]; *Stanev* (n 83) [130]–[131]; *Reference* (n 2) [159]–[167].

95
*Reference* (n 2) [164], [176]; *Stanev* (n 83) [130]; *Stankov* (n 91) [89]; *Sykora v Czech Republic* App no 23419/07 (ECtHR, 22 November 2012); *Lashin v Russia* App no 33117/02 (ECtHR, 22 January 2013); Anna Arstein-Kerslake, *Restoring Voice to People with Cognitive Disabilities: Realizing the Right to Equal Recognition before the Law* (CUP 2017) ch 6.

96
*Nielsen v Denmark* (1989) 11 EHRR 175; *Reference* (n 2) [149]–[150], [156].

97
*In re D* (n 19) [42] (Lady Hale).

98
*Reference* (n 2) [85], [93]–[94].

99
*Storck* (n 5) [77]; *HL* (n 10) [93]; Practice Statement (n 4).

100
*R (Ullah) v Special Adjudicator* [2004] UKHL 26, [2004] 2 AC 323 [20] (Lord Bingham); *Reference* (n 2) [46]; *In re G* (n 69).

101 European Convention on Human Rights, art 14; *Glor v Switzerland* App no 13444/04 (ECtHR, 30 April 2009); *Thlimmenos v Greece* (2001) 31 EHRR 15; *R (SC) v Secretary of State for Work and Pensions* [2021] UKSC 26, [2022] AC 223.

102
*De Wilde* (n 8) [65]; *MM* (n 8); *Buzadji* (n 8); *Williams* (n 8); *In re T* (n 46) [161]; *Re SA* (n 47); *DL v A Local Authority* [2012] EWCA Civ 253; *M v Ukraine* (n 90) [77].

103 Draft Code (n 3) paras 2.12–2.13; Joint Submissions (n 19) para 57. The first instance decisions bear this out: see *In re Mustafa* [2026] EWHC 2159 (Fam), in which the subjective limb was applied with a light touch to a boy with mental disabilities, discussed in Craig Wells, ‘The Veneer of Contentment: Mustafa, Salford and the Gaps the Deprivation of Liberty Reference Left Behind’ (UK Constitutional Law Blog, 19 October 2026) <https://ukconstitutionallaw.org/2026/10/19/craig-wells-the-veneer-of-contentment-mustafa-salford-and-the-gaps-the-deprivation-of-liberty-reference-left-behind/> accessed 25 October 2026.

104 Ruby Reed-Berendt and Agomoni Ganguli-Mitra, ‘The Relationship between Capacity and Credibility: Implications for Epistemic Injustice’ (2025) 33 Medical Law Review fwaf039, drawing on Kristie Dotson, ‘Conceptualizing Epistemic Oppression’ (2014) 28 Social Epistemology 115.

105
*Reference* (n 2) [198]–[199]; *Re SM (Deprivation of Liberty: Severely Disabled Child)* [2024] EWHC 493 (Fam), [2024] 2 FLR 896 [42]–[43] (Lieven J).

106
*HL* (n 10) [90].

107 Convention on the Rights of Persons with Disabilities, arts 12(2) and 14(1).

108
*R (SC)* (n 100); *JB* (n 7) [120].

109
*ET v Moldova* [2024] ECHR 858 [74]; Joint Submissions (n 19) para 42.

110 General Comment No 1 (n 65) paras 15–19; Arstein-Kerslake (n 95); Series (n 61).

111
*Cheshire West* (n 1) [57].

112 Peter Bartlett, ‘Re-thinking the Mental Capacity Act 2005: Towards the Next Generation of Law’ (2023) 86 Modern Law Review 659, 660, 687; Stavert and McKay (n 11) 4.

113
*Reference* (n 2) [203]–[204].

114 ibid [205].

115 ibid [75]–[77].

116
*Reference* (n 2) [83], [97]; *In re Dalton* [2023] UKSC 36, [2025] AC 235 [47]; *Horton v Sadler* [2006] UKHL 27, [2007] 1 AC 307 [29] (Lord Bingham); Practice Statement (n 4).

117
*Reference* (n 2) [99]–[104], quoting Series (n 61) 186; Law Commission, *Mental Capacity and Deprivation of Liberty* (Law Com No 372, 2017) paras 2.23–2.24.

118
*Rochdale Metropolitan Borough Council v KW* [2014] EWCOP 45, [2015] 2 FCR 244 (Mostyn J); *Reference* (n 2) [117].

119
*Birmingham City Council v D* [2016] EWCOP 8 [138] (Keehan J); *Re NRA* [2015] EWCOP 59 [25] (Charles J).

120
*N v ACCG* [2017] UKSC 22, [2017] AC 549 [35]; Giles Birchley and Aoife Finnerty, ‘Freedom Isn’t Free: Resource Limits on Person-Centred Best Interests Decisions under the Mental Capacity Act’ (2026) 34 Medical Law Review fwag001.

121 Joint Submissions (n 19) para 4; recorded at *Reference* (n 2) [31].

122 Wise and Spurrier, ‘A Gilded Cage is Still a Cage’ (2014) 164 New Law Journal 7607, 7.

123
*Re BJ (Incapacitated Adult)* [2009] EWHC 3310 (Fam) [2], [10]; *Reference* (n 2) [136], [140]–[141].

124
*De Wilde* (n 8) [73], [76]; *HL* (n 10) [140]; *Reference* (n 2) [7].

125
*Reference* (n 2) [53(v)], [191].

126 Draft Code (n 3) para 2.13; *Reference* (n 2) [16]; Joint Submissions (n 19) para 57.

127
*Austin v Commissioner of Police of the Metropolis* [2009] UKHL 5, [2009] 1 AC 564 [61] (Lord Neuberger).

128 Paula Case, ‘Dangerous Liaisons? Psychiatry and Law in the Court of Protection: Expert Discourses of “Insight” (and “Compliance”)’ (2016) 24 Medical Law Review 360, 376–377.

129 Joint Submissions (n 19) para 53.

130 Donnelly (n 59) 327.

131 Joint Submissions (n 19) paras 38–39.

132 Reed-Berendt and Ganguli-Mitra (n 104).

133 Joint Submissions (n 19) paras 46–48; the court acknowledged this evidence: *Reference* (n 2) [54]–[55].

134 Joint Submissions (n 19) para 49; see also *Re RD* [2016] EWCOP 49 [88]–[107], discussed ibid para 45.

135 Joint Submissions (n 19) para 7; European Convention on Human Rights, art 34; cf *Reference* (n 2) [49], citing *R (AB) v Secretary of State for Justice* [2021] UKSC 28, [2022] AC 487 [56]–[57].

136
*HL* (n 10) [9]; *Re BJ* (n 123) [14]; *Austin* (n 127) [44] (Lord Walker); Series (n 61) 208–210.

137
*Reference* (n 2) [26], [52]–[53].

138
*Ferreira* (n 66) [89], [93]; cf *Reference* (n 2) [114]–[116].

139
*Reference* (n 2) [107]–[113]; Joint Committee on Human Rights, *The Right to Freedom and Safety: Reform of the Deprivation of Liberty Safeguards* (2018 HC 890, HL 161) para 44; Series (n 61) 182–183; for the Official Solicitor’s concerns, *Reference* (n 2) [34].

140
*Cheshire West* (n 1) [57].

141 Dougherty, ‘Informed Consent, Disclosure, and Understanding’ (n 49) 139–140; Donnelly (n 59) 327.

142
*Reference* (n 2) [75]–[77], [205]; *Cheshire West* (n 1) [46].

